# Diagnosis and Management of Neuropathic Pain in Spine Diseases

**DOI:** 10.3390/jcm12041380

**Published:** 2023-02-09

**Authors:** Joanna Bielewicz, Maciej Kamieniak, Michał Szymoniuk, Jakub Litak, Wojciech Czyżewski, Piotr Kamieniak

**Affiliations:** 1 Department of Neurology, Medical University of Lublin, Jaczewskiego 8, 20-954 Lublin, Poland; 2 Department of Neurosurgery and Pediatric Neurosurgery, Medical University of Lublin, Jaczewskiego 8, 20-954 Lublin, Poland; 3 Department of Clinical Immunology, Medical University of Lublin, Chodźki 4A, 20-093 Lublin, Poland; 4 Department of Didactics and Medical Simulation, Medical University of Lublin, Chodźki 4, 20-093 Lublin, Poland

**Keywords:** neuropathic pain, neuralgia, pathophysiology, therapeutics, spinal diseases

## Abstract

Neuropathic pain is generally defined as a non-physiological pain experience caused by damage to the nervous system. It can occur spontaneously, as a reaction to a given stimulus, or independently of its action, leading to unusual pain sensations usually referred to as firing, burning or throbbing. In the course of spine disorders, pain symptoms commonly occur. According to available epidemiological studies, a neuropathic component of pain is often present in patients with spinal diseases, with a frequency ranging from 36% to 55% of patients. Distinguishing between chronic nociceptive pain and neuropathic pain very often remains a challenge. Consequently, neuropathic pain is often underdiagnosed in patients with spinal diseases. In reference to current guidelines for the treatment of neuropathic pain, gabapentin, serotonin and norepinephrine reuptake inhibitors and tricyclic antidepressants constitute first-line therapeutic agents. However, long-term pharmacologic treatment often leads to developing tolerance and resistance to used medications. Therefore, in recent years, a plethora of therapeutic methods for neuropathic pain have been developed and investigated to improve clinical outcomes. In this review, we briefly summarized current knowledge about the pathophysiology and diagnosis of neuropathic pain. Moreover, we described the most effective treatment approaches for neuropathic pain and discussed their relevance in the treatment of spinal pain.

## 1. Introduction

Neuropathic pain is generally defined as a non-physiological pain experience caused by damage to the nervous system [1]. It can occur in response to a given stimulus, independently of that stimulus, or spontaneously. Pain sensations reported by patients are usually referred as firing, burning or throbbing [2]. The etiology may be primary, when the pathological process involves the nervous tissue, or secondary, when the surrounding tissues are involved [3]. In many cases, it is difficult to distinguish the neuropathic component of pain reported by the patient, so neuropathic pain often occurs unnoticed by a physician. However, it is estimated that it affects about 7–8% of the general population [4].

Neuropathic pain is a clinical description, not a diagnosis. The states characteristic for the phenomenon of neuropathic pain are hyperalgesia, which is an excessive reaction to a given pain stimulus, and allodynia, defined as a feeling of pain resulting from the action of a stimulus that should not normally cause it [5]. Both suggest neuropathic pain, but are not necessary for its diagnosis.

There are many divisions depending on the mechanisms that lead to pain. Thus, there is central pain, when the damage leading to its occurrence is to the brain or spinal cord, and peripheral pain, originating from peripheral nerves, plexus and roots [6]. Interestingly, when the spinal cord is injured, up to half of the patients may experience neuropathic pain, while peripheral nerve injury is associated with a higher prevalence (approximately 73%) [7,8]. Another division distinguishes the various natures of the cause of the phenomenon, for example, mechanical, inflammatory, vascular or metabolic [9,10]. The same condition can cause severe pain in one patient without causing any in others, but the reasons for this differentiation remain unknown [2].

In the course of spine disorders, pain symptoms commonly occur [11,12]. According to available epidemiological studies, a neuropathic component of pain is often present in patients with spinal diseases, with a frequency ranging from 36% to 55% of patients [13,14]. Therefore, the use of therapeutic methods effective for neuropathic pain may be beneficial in the treatment of pain caused by spine disorders. However, molecular mechanisms participating in the pathomechanism of neuropathic pain are not fully understood, which may be the cause of the insufficient efficacy of the current treatment. Thus, to better investigate the pathomechanism of neuropathic pain and improve the effectiveness of therapy, an inverse translational approach has been recently introduced [15].

In this review, we briefly summarized current knowledge about the pathophysiology and diagnosis of neuropathic pain. Animal models of neuropathic pain, which are especially important today in the era of inverse translational approaches in neuropathic pain research, have also been presented. Moreover, we described the most effective treatment approaches for neuropathic pain and discussed their relevance in the treatment of spinal pain.

## 2. Pathophysiology of Neuropathic Pain

The mechanisms leading to the occurrence of pain are complex. Damage to the somatosensory system essentially leads to a loss of sensation, which is a so-called negative sensor sign [1]. However, some of the nerves may remain undamaged or partially damaged and are responsible for the conduction of not necessarily correct sensory feelings. Therefore, multiple processes within the lesion can simultaneously cause positive signs, of which ongoing pain is the most prominent one [1]. These phenomena may be caused either by increased neuronal transmission or by the weakening of the inhibitory processes, and also as a result of both of these processes. There is a frequent coexistence of both negative and positive sensory phenomena [16]. Understanding the pathologies responsible for the development of neuropathic pain is crucial in selecting the appropriate therapy.

The changes leading to the development of neuropathic pain include all levels of sensor transmission, the peripheral structures and their surroundings, as well as the central nervous system, and the processes on individual levels are sometimes slightly different. At the peripheral level, damage may be the cause of increased sensitization; secondarily, it may lead to ectopic impulses generation due to both degeneration in the affected area and subsequent regeneration [17]. These impulses, conducted via the normal nociceptive pathways, result in projected pain, which is a feeling of pain in the part of the body where the damage has occurred [18]. In addition, central sensitization and reorganization of the sensory fields can take place in the central nervous system, often as a result of microglia activity and excessive neurotransmission [17]. These changes, also sometimes manifested as projected pain, can secondarily cause further pathological processes in the more rostral structures of the nervous system [19]. Therefore, peripheral lesions leading to neuropathic pain lead to both peripheral and central changes, while lesions in the central nervous system are only characterized by the appearance of central changes.

### 2.1. Peripheral Mechanisms

Ectopic potentials seem to be the main mechanism responsible for the induction of peripheral neuropathic pain [20]. The spontaneous generation of potentials may be caused by a change in the expression of sodium channels in injured nerves [21]. The intact nerves necessary for conducting such signals are reportedly often subject to pathological sensitization within damaged tissue, where the expression of various growth factors that act on intact nerves is enhanced [22]. Ectopic generated potentials are conducted along the normal nerve pathways to the brain, which interprets them as pain from the territory of the body normally innervated by a given nerve, which underlies the phenomenon of projected pain [22].

Such stimulations conducted through the normal nociceptive pathways may induce use-dependent synaptic transmission [23]. This physiological phenomenon may be the first cause of central sensitization secondary to peripheral changes. The remaining reasons result from the above-mentioned over-expression of growth factors leading to changes in expression in the dorsal root ganglion and the axonal transport of chemokines that stimulate the spinal microglia [24]. Ultimately, central sensitization leads to increased activity of neurons receptive to normal or subliminal stimuli [23].

Therefore, in peripheral damage, pain is the result of both signals from the damaged tissue and conducted along the normal conduction pathways, and secondary changes in these pathways, also at the central level.

### 2.2. Central Mechanisms

The aforementioned central sensitization may be secondary to peripheral changes or result directly from pathology in the central nervous system. Synapses in the afferent pain pathways are subject to use-dependent plasticity [23]. These processes are similar to long-term potentiation and long-term depression in the hippocampus [25]. In contrast to the hippocampus, in the spinal cord, however, long-term potentiation-like phenomena can be induced by relatively low-frequency inputs, such as stimuli normally flowing into nociceptive neurons [26]. Many transmitters are involved in the formation of central sensitization and the loss or impairment of their functions may enhance central sensitization. Of importance are N-methyl-D-aspartate (NMDA) receptors for glutamate and neurokinin 1 (NK1) receptors for substance P, which are secreted presynaptically in nociceptive neurons [27]. Glutamate receptors of the alpha-amino-3-hydroxy-5-methyl-4-isoxazolepropionate (AMPA) type are highly regulated in the spinal cord plasticity processes [28]. Pain transmission is also regulated by a network of interneurons, some of which have inhibitory action through glycine or gamma-aminobutyric acid (GABA) [29].

Similar ectopic impulses can occur in the central nervous system, as in the case of peripheral pathologies. For example, bursting activity is observed in the somatosensory part of the thalamus and some animal models even suggest that this activity may be generated in the thalamus itself [30]. This is due to electrophysiological changes in the central nervous system, such as the upregulation of voltage-gated sodium channels [30]. Additionally, the decreased inhibition on the part of the GABAergic fibers may contribute to these disorders [31].

The perception of pain is physiologically regulated by descending fibers, both inhibitory and excitatory [19]. The role of rostroventral medulla is important here. Disorders of these mechanisms are also considered to be one of the central mechanisms responsible for the phenomenon of neuropathic pain, and although the reduced inhibition may play an important role in the phenomenon of hyperalgesia, it is suggested that the role of increased descending facilitation is much more important [32,33,34].

Another aspect observed in the case of neuropathic pain is the reorganization of the receptive field; however, it is not certain whether these mechanisms are one of the causes or a consequence of the existing pathology [35].

## 3. Animal Models of Neuropathic Pain

Neuropathic pain is a set of phenomena responsible for the occurrence of pain and other sensory pathologies that are inadequate to the stimulus that causes them, or even appear spontaneously regardless of this stimulus [36]. It includes such phenomena as hyperalgesia and allodynia [5]. These phenomena result, as already described, from peripheral and central causes, the mutual correlations of which depend on the location and nature of the primary pathology. Research on the nature of neuropathic pain is carried out in animal models that are highly differentiated in terms of the location of the damage on different levels of the afferent pathways from the peripheral nerve, through the spinal nerve to the spinal cord, and the type of damage, including traumatic, metabolic or toxic changes. The reconstruction of various disease states in animals, in which the phenomenon of neuropathic pain occurs, helps to understand the nature of the phenomenon, understand the role of particular pathologies that comprise the clinical picture, and search for therapeutic solutions that allow for effective treatment. It should be emphasized, however, that a large proportion of animal models only reflect single aspects, while clinically occurring neuropathic pain is a much more complex and diverse phenomenon, and a patient suffering from it may actually represent several phenomena simultaneously, composing a uniform picture of neuropathic pain. In all animal models described below, rats are the species of choice.

### 3.1. Chronic Constriction Injury

An important aspect of neuropathic pain is incomplete damage to the nerve structures. Although some of the fibers are completely damaged, the remaining partially damaged or even intact ones may conduct the impulses responsible for the occurrence of the discussed phenomena [22]. In the chronic constriction injury (CCI) model, this is achieved by placing loose sutures on the sciatic nerve [37]. As a result, some of the fibers degenerate, but the remaining, mostly afferent, fibers remain functional. Moreover, the use of chromic catgut provides additionally inflammatory components [38]. A few days after such surgery, symptoms typical of neuropathic pain can be observed in rats.

The phenomena of mechanical, chemical and thermal hyperalgesia and thermal allodynia are monitored and measured using special devices. Moreover, spontaneous pain sensations are demonstrated. Hyperalgesia lasts about several months and is often followed by hypoalgesia [39].

The undoubted advantage of this method is the wide spectrum of phenomena typical for neuropathic pain, which it causes, reflecting the potential clinical condition as effectively as possible. This makes the model a good field for testing and developing therapies. In this way, the effects of tricyclic antidepressants, such as amitriptyline, which reduces thermal and mechanical hyperalgesia, or clomipramine and desipramine, which act on mechanical hyperalgesia, have been proven [40,41]. Also in humans, antidepressants have proved to be effective as components of the treatment of diabetic or even post-herpetic neuropathy [42]. The action of antiepileptic drugs such as gabapentin has been similarly proven [43]. In a recent study on the CCI rat model, the anti-inflammatory and analgesic effects were demonstrated by quercetin, a plant flavonol [44]. Moreover, it has been observed that quercetin acts through the inhibition of the MAPK signaling pathway and stimulation of the AMPK pathway. However, the CCI model of neuropathic pain presents some imperfections, such as uncontrollable tension of the sutures (it may influence the amount of injured nerve fibers), demonstration of hyperalgesia (it is not presented by clinical neuropathic pain), and less intensified allodynia compared with other NP models [45].

### 3.2. Segmental Spinal Nerve Ligation

The difference between segmental nerve ligation (SNL) and CCI is that in SNL, not only one nerve is damaged, but the ligation takes place on a given segment of the spinal cord (L5 or L5 and L6 are injured, whereas the L4 spinal nerve remains intact) [45]. As a result, the sciatic nerve has both damaged (L5 and L6) and undamaged (L4) nerve fibers. Moreover, this NP model requires extensive surgical procedure exposing spinous processes at the L4-S2 levels [45]. Long-lasting ongoing pain, heat hyperalgesia, cold allodynia and mechanical allodynia have been observed after SNL [46].

SNL is widely used in preclinical research on the treatment of neuropathic pain. Drugs such as carbamazepine, gabapentin, felbamate and pregabalin have been studied [47]. Recently, adipose tissue-derived stem cells demonstrated effective action against the cold allodynia in a rat SNL model [48]. Similarly, the method was used for the purpose of a deeper understanding of the pathophysiology of the phenomenon: for example, the redistribution of sodium channels in undamaged axons [49]. Moreover, the SNL model showed that in central sensitization mechanisms, the CXCL12/CXCR4 signaling axis may be involved, which constitutes the potential aim of neuropathic pain treatment [50]. Nevertheless, muscle damage during the surgery (which may influence the pathologic mechanism) and a demanding surgical approach with avoiding any injury of the L4 nerve represent the main disadvantages of the SNL model [45].

### 3.3. Partial Sciatic Nerve Ligation

The partial nerve ligation (PNL) model is a modified version of the CCI where the dorsal part of the rats' nerve thickness (one third to one half) is ligated [51]. The model results in mechanical hyperalgesia and allodynia, and signs of spontaneous pain. These symptoms occur within hours after ligation and remain for about 7 months [45]. The PNL model imitates contusion rather than compression of the nerve [45].

The effectiveness of gabapentin against central sensitization and the prevention of thermal hyperalgesia by amitriptyline were proven in studies using PNL animal models [52,53]. Furthermore, icariin—a natural flavonoid obtained from the Epimedium species—investigated on the PNL model demonstrated the alleviation of neuropathic pain through anti-apoptotic and anti-inflammatory features [54]. Moreover, easy surgical procedure and high reproducibility are the main benefits of the PNL model [45].

### 3.4. Spared Nerve Injury

In the spared nerve injury (SNI) model, the sciatic nerve is exposed and the tibial nerve, as well as the common peroneal nerve, are sectioned [55]. The sural nerve remains intact [56]. In this model, mechanical allodynia and hyperalgesia, and cold allodynia as well as heat hyperalgesia, were observed 2–3 days after damage [55,57]. The mentioned symptoms disappear after at least 15 months in the case of rats and 30 days in the case of mice [45].

Based on this model, the relieving effect of gabapentin, amitriptyline and opioids has been proven [58,59,60]. The main advantages of the SNI model include high reproducibility, an easy surgical procedure, a short duration of pain symptoms, and a lack of influence on animal’s daily activity [61]. On the other hand, compared with other models, a decreased level of local inflammation is observed in the SNI model [56]. A recent study by He et al. developed a modified SNI model through injuring the sural and common peroneal nerves and keeping intact the tibial nerve [62]. This model, defined by authors as SNIt, exhibits less motor dysfunction compared with the traditional SNI model.

### 3.5. Diabetic Neuropathy

Several models of diabetic neuropathy have been developed because neuropathic pain is a fairly common component of it [63]. These include chemically, genetically and diet-induced diabetes. Diabetes induced by injecting the rats with pancreatic B-cell toxic compound streptozotocin or alloxan, as well as that induced by a high-fat diet, corresponds to a disease with a normal genetic background [64,65,66]. Thermal and mechanical hyperalgesia, and mechanical and chemical allodynia in the chemical model, as well as mechanical allodynia and thermal hyperalgesia in the case of diet-induced diabetes, have been observed here. In genetically determined diabetes, thermal hyperalgesia and mechanical allodynia have been observed [67].

Diabetic models have been used to study the effects of a number of analgesics, including pregabalin, diclofenac, TCAs and morphine, the latter two being effective [68,69,70,71].

### 3.6. Spinal Cord Injury and Neuropathic Pain

Spinal cord injury is often manifested by the coexistence of neuropathic pain [72]. Due to the multitude of mechanisms leading to it, there are many models that reflect the clinical condition of the patient, including spinal cord hemisection, spinal cord ischemia and spinal contusion [73,74,75].

Spinal cord hemisection corresponds to Brown-Sequard syndrome, and thermal and mechanical allodynia have been observed [73].

Ischemia of a specific segment of the spinal cord is achieved through the application of photo-reactives and the use of a laser. The reaction in the rats' vessels leads to occlusion followed by ischemia of the desired segment. Mechanical allodynia was among others observed in the research [74]. This model was used to determine the clinical efficacy of drugs such as morphine, tocainide, baclofen, pentobarbital and carbamazepine [76,77,78,79,80].

The spinal contusion model is achieved by dropping weights onto a fixed open spinal cord, which results in specific degrees of damage depending on the height from which the weights are dropped. The phenomena observed in this model are mechanical and thermal allodynia, and the model was used to deepen the knowledge of the pathophysiology of neuropathic pain with an indication of central pain [75]. Moreover, gabapentin has been proven to act against allodynia [81].

### 3.7. Visceral Pain Models

Models involving visceral pain include interstitial cystitis, endometriosis and prostatitis, the former of which is the most common in research [82,83,84]. Pain is often caused by the intraperitoneal administration of cyclophosphamide, which accumulates and undergoes metabolism, leading to inflammation and pain within 15–30 min of administration [85]. Another way to induce interstitial cystitis is by administering a pseudorabies virus, which is injected into the abductor caudalis dorsalis muscle [86]. Mast cell inflammation in this model suggests a strong association between neurogenic pain and inflammation.

## 4. Diagnosis and Frequency of Neuropathic Pain in Spine Diseases

Chronic pain is the primary symptom of patients suffering from spine diseases. Pain can vary in nature and severity depending on the mechanisms by which it is triggered. Although the nociceptive mechanism is the basic one, the neuropathic component sometimes seems to be equally important and may significantly affect the treatment and outcome of patients [87]. It should be noted that distinguishing between chronic inflammatory or nociceptive pain and neuropathic pain very often remains a challenge [88]. This is due to many factors, including the broad subjectivization of patients’ pain sensations, various dynamics of pain progression, the lack of an obvious clinical test to assist the diagnosis, as well as the imprecise definition of neuropathic pain [87].

A detailed anamnesis and medical examination increase the chance of a correct diagnosis. While nociceptive pain is usually sharp and rushing, neuropathic pain is stinging, tingling, numb, stabbing, and is accompanied by decreased sensitivity in the affected area [89,90]. There are no specific patterns of its course or triggers. It can appear spontaneously or be induced; sometimes it lasts constantly; other times it can be paroxysmal [90]. The occurrence of neuropathic pain may be indicated by the presence or history of a disease that could cause nerve damage. Moreover, the nerve damage confirmed by neurophysiological, neuroimaging, or during the surgery, in correlation with the overall assessment of the patient, allows for an almost certain diagnosis. The absence of a sensory disturbance and a good response to typically used analgesics make the diagnosis of neuropathic pain unlikely [90]. Various scales and questionnaires have been developed to help assess pain in patients and make the diagnosis of neuropathic pain, including the Leeds Assessment of Neuropathic Symptoms and Signs score (LANSS), the Northwick Park Neck Pain Questionnaire (NPQ), DN4 (Douleur Neuropathique 4 Questions) questionnaire, and painDETECT questionnaire [91,92].

Due to the aspects described above, neuropathic pain is often underdiagnosed in patients with spinal diseases. In the general population, the incidence of neuropathic pain is estimated to be from 0.82% to approximately 3%, while the overall incidence among chronic pain patients is estimated at 6.9% [93]. There are many studies assessing the incidence of neuropathic pain in patients with spine disease. For instance, Kim et al. described a group of 1109 patients who qualified for lumbar spine surgery, of whom 404 were diagnosed with neuropathic pain using the LANSS scale, which accounted for 36.4% of all assessed patients [94]. A very similar result on a smaller group of patients with lumbar spine stenosis was reported by Park et al. diagnosing neuropathic pain in 31 out of 86 patients (36%). However, this study divided patients complaining of radicular pain and neurogenic claudication, and in the former, the neuropathic component was much more frequent, occurring in 24 of them, which constituted 63.4%, while the remaining 7 with neurogenic claudication accounted for only 15.6%. It should be noted that the vast majority (78, which constituted 90.7%) of patients presented both root pain and claudication pain, and the above-mentioned division was made on the basis of the dominant component [95]. El Sissi et al. described the incidence of neuropathic pain in 55% of 1134 patients with chronic back pain [14]. Yamashita et al. reported a 53.5% frequency in patients with diseases of the spine [13]. Hassan et al. presented the prevalence of NP in 41% of the examined patients with back pain [96]. Attal et al. described the prevalence of neuropathic pain in patients, implying a higher occurrence correlated with lumbar spine stenosis then other causes [97,98] (Table 1).

The studies presented above prove how common the phenomenon of neuropathic pain is, which shows its clinical significance. As mentioned also, neuropathic pain is not usually responsive to standard NSAID analgesia. The response to drugs affecting opiate receptors is also sometimes limited. Antidepressants seem to be much more effective in the treatment of spinal diseases with a neuropathic component, including, for example, selective serotonin norepinephrine reuptake inhibitors (SSNRIs). Moreover, calcium channel α2-δ ligands and topical lidocaine might be useful [99,100,101,102,103]. This is one of the reasons why the diagnosis of neuropathic pain in diseases of the spine may play a key role in the selection of an effective therapy and contribute to a better outcome, especially in the treatment of patients with chronic pain not relieved by conventional therapy.

## 5. Treatment Strategies for Neuropathic Pain

In recent decades, a plethora of therapeutic methods for neuropathic pain have been developed and investigated. In the following sections, we describe currently recommended and emerging methods of treatment for neuropathic pain, and discuss their usefulness for the neuropathic component of chronic low back pain.

### 5.1. Pharmacotherapy

According to the recently updated (2020) French guidelines for the treatment of neuropathic pain, gabapentin, serotonin and norepinephrine reuptake inhibitors (SNRIs), and tricyclic antidepressants (TCAs), constitute first-line therapeutic agents [104]. On the other hand, pregabalin, tramadol, botulinum toxic A, capsaicin patches, combination therapies and psychotherapy constitutes are recommended as second-line therapy. Among third-line treatments, pharmacological agents such as strong opioids have also been considered. Moreover, gabapentinoids, SNRIs and TCAs were also proposed by NeuPSIG as first-line therapeutic agents [105]. These recommendations show the continuing major significance of pharmacotherapy in the management of neuropathic pain (Table 2).

#### 5.1.1. Gabapentinoids

Pregabalin and gabapentin constitute the most common gabapentinoids prescribed in order to alleviate neuropathic pain in adults [139]. The gabapentinoids mechanism of action relies on binding to the α2-δ subunit of presynaptic voltage-gated Ca^2+^ channels in the dorsal horn of the spinal cord [106]. This leads to a decrease in the release of neurotransmitters, such as norepinephrine, glutamate and substance P. Consequently, that molecular mechanism is responsible for their analgesic effects [139]. Both gabapentin and pregabalin have demonstrated successful outcomes in the treatment of pain caused by postherpetic neuralgia, spinal cord injury, phantom limb syndrome and diabetes-induced neuropathy [108]. Most common complications observed in patients treated with gabapentionoids include peripheral edema, somnolence, dizziness, xerostomia and obesity [107].

Numerous scientific associations have recommended gabapentinoids as first-line drugs for neuropathic pain [104,105,140]. Moreover, the use of gabapentin and pregabalin for this purpose has been approved by the Food and Drug Administration (FDA) [108]. However, in a recent update of French guidelines, pregabalin was downgraded to a second-line treatment due to the risk of misuse and addiction, and a higher risk of respiratory depression in combination with opioids reported by the latest studies [129,141]. Moreover, a recent randomized controlled trial demonstrated that pregabalin was less safe and less effective in relieving sciatica compared with gabapentin [142].

Although pregabalin and gabapentin have demonstrated efficacy in the treatment of many types of neuropathic pain, their effectiveness in the treatment of neuropathic pain associated with spine diseases is not evident. Gabapentin has demonstrated significant improvements in sensory deficits, relieving pain and neurogenic claudication associated with lumbar spinal stenosis [143,144]. However, a recent meta-analysis did not recommended anticonvulsants as a first line treatment for lumbar spinal stenosis causing neurogenic claudication [145]. Regarding the use of gabapentinoids for sciatica, clinical trials evaluating their effectiveness in sciatica have showed unsatisfactory results [146,147,148]. A recent meta-analysis indicated that pregabalin and gabapentin did not provide improvement in sciatica compared with placebo and suggested their clinical use should not be supported for this purpose [149]. However, sciatica is characterized by a combination of nociceptive and neuropathic pain, where nociceptive pain constitutes the main component [150]. Therefore, gabapentionids, which cannot alleviate the nociceptive component of pain, may be ineffective for the management of sciatica.

A novel selective α2-δ ligand, mirogabalin, has shown safety and effectiveness in the management of postherpetic neuralgia and diabetic neuropathic pain [109]. Recently, mirogabalin has been approved for the treatment of peripheral neuropathic pain in Japan [151]. A retrospective study by Kim et al. evaluated mirogabalin in relieving neuropathic pain in 60 patients with lumbar spine disease [152]. Mild adverse effects in 17 patients and a significant improvement in leg pain symptoms were observed. Moreover, mirogabalin also alleviated low back pain and sleep disturbance. However, this study has some major limitations, such as no control group, a small study population and a retrospective design. In a multicenter randomized open-label study entitled MiroTAS, the addition of mirogabalin to the NSAIDs treatment of peripheral neuropathic pain related to lumbar spinal stenosis improved patients’ quality of life and decreased pain intensity compared with NSAIDs alone [153]. Although somnolence and dizziness were common adverse effects in the study group (30% and 25.5% respectively), the addition of mirogabalin to NSAIDs was considered a generally safe combination. Moreover, in another study, a decrease in somnolence and dizziness frequency was observed in patients who switched from pregabalin to mirogabalin (from 12.2% to 7.3% and from 14.6% to 4.9% respectively) [110]. The above findings suggest that mirogabalin may be a safer and similarly effective therapeutic approach for peripheral neuropathic pain in patients with spine diseases compared with other gabapentinoids, although further studies are necessary.

#### 5.1.2. Antidepressants

Tricyclic antidepressants (TCAs) such as amitriptyline have been used as a treatment for neuropathic pain for many years and have been recommended as first-line drugs [140,141,154]. Their effectiveness has been proven in the treatment of postherpetic neuralgia, central postpartum pain, pain after spinal cord injury and nerve injury [105,108]. Recent studies have reported that TCAs also exhibit antitumoral effects and may be useful for the treatment of neuropathic pain associated with neoplastic disorders [155]. The exact mechanism underlying the analgesic properties of TCAs remains unclear. TCAs may inhibit neuropathic pain through increasing noradrenaline, which acts on α2-adrenergic receptors in the dorsal horn of the spinal cord. TCAs can also increase the level of noradrenaline in the locus coeruleus, which stimulates an impaired descending noradrenergic inhibitory system [111]. It is worth noting that the use of TCAs is contraindicated for patients suffering from cardiovascular disease, prostate hypertrophy and glaucoma [108,112]. Furthermore, cognitive impairment and walking disturbances may occur during therapy with TCAs [112]. Other often adverse effects reported in neuropathic pain studies in patients receiving TCAs include urinary retention, constipation, dry mouth and orthostatic hypotension [113]. However, the effects of long-term TCAs administration are not fully investigated in the available studies regarding the treatment of neuropathic pain, whereas severe withdrawal syndrome is a known long-term complication of therapy with TCAs [156].

According to a recent review, the use of TCAs in the treatment of low back pain can be successful [157]. However, the optimal dose of TCAs was not indicated in this study and was determined based on patient tolerance. Moreover, the low daily dose (10 mg) of TCAs such as amitriptyline or nortriptyline demonstrated the alleviation of leg and back pain in patients with lumbar spinal stenosis [158]. A meta-analysis by Ferreira et al. showed that TCAs were ineffective in reducing pain in patients with low back pain, but decreased pain intensity in patients with sciatica at a minimum of 2 weeks of follow-up [159]. However, the certainty of evidence obtained in this meta-analysis was low or even very low due to a limited number of patients, a high risk of bias, and imprecise estimates present in the included studies.

Duloxetine and venlafaxine are the most studied serotonin and norepinephrine reuptake inhibitors (SNRIs) for neuropathic pain treatment. These agents have been approved by the FDA for the treatment of fibromyalgia, diabetic neuropathy and back pain [114,115,116]. Due to the similarity of effects exerted by SNRIs to TCAs ones, their analgesic properties possibly result from increasing the level of noradrenaline in the dorsal horn of the spinal cord and locus coeruleus [111]. Common adverse reactions caused by SNRIs application include lack of appetite, constipation, dry mouth, anxiety, hyperhidrosis and nausea [117]. Moreover, SNRIs should be used with caution in patients with cardiovascular disease [160]. It is worth noting that the use of venlafaxine is associated with withdrawal syndrome [161].

In the treatment of chronic low back pain, the effectiveness of duloxetine is comparable to other oral pharmacological agents [162,163]. In a double-blind randomized controlled trial investigating the use of duloxetine in chronic low back pain with the neuropathic component, duloxetine significantly decreased pain symptoms by an average of 32% compared with placebo [164]. According to current studies, the optimal daily dose of 60 mg of duloxetine demonstrates the highest effectiveness in relieving pain, with the risk of complication reduced to a minimum [165]. Moreover, a recent retrospective study demonstrated the effectiveness of duloxetine in reducing postsurgical chronic neuropathic disorders in 19 of 24 patients after spine or spinal cord surgeries [166]. However, due to retrospective character and a limited number of patients in this clinical trial, the optimal use of duloxetine in patients with spine surgery-induced neuropathic pain should be investigated in further, well-designed randomized studies.

#### 5.1.3. Topical Agents

Five-percent lidocaine patches have been indicated as a second-line agent by NeuPSIG recommendations and as a first-line agent for neuropathic pain according to recent French guidelines [105,140,141]. Lidocaine's mechanism of action relies on blocking voltage-gated Na^+^ channels, which decrease spontaneous nerve discharge [120]. Topical lidocaine is considered as the safest treatment alternative for neuropathic pain, and skin irritation constitutes its main adverse effect [119]. Numerous studies have demonstrated the efficacy of topical lidocaine in the treatment of postherpetic neuralgia, postsurgical chronic neuropathic pain and diabetic peripheral neuropathy [118]. For the treatment of pain caused by spine diseases, lidocaine can be administered both in the form of patches and paraspinal injections. Most of the studies published to date concerning lidocaine patches for chronic low back pain present little evidence due to their uncontrolled and open-label character [167]. A randomized controlled trial by Hashmi et al. demonstrated the non-superiority of a 5% lidocaine patch in the alleviation of chronic back pain compared with a placebo [168]. Therefore, the clinical efficacy of lidocaine patches in the treatment of low back pain is questionable. On the other hand, paraspinous lidocaine injections in combination with standard treatment demonstrated better outcomes in relieving low back pain compared with standard treatment alone, without an increased risk of adverse effects [169].

Topical capsaicin is generally recommended as a second-line drug, as neuropathic pain exerts its analgesic action through the modulation of transient receptor potential cation channel vanilloid subfamily member 1 (TRPV1), which is involved in the pain modulation mechanisms [105,140,141,170]. Similar to topical lidocaine, capsaicin is absorbed into the circulation at a minimum, and transient skin reactions dominate the adverse effects of this agent [121]. However, its potential long-term influence on nerve fibers is unknown [112]. The efficacy of 8% capsaicin patches was established in diabetic peripheral neuropathy, but beneficial outcomes were also reported in the treatment of postherpetic neuralgia and HIV neuropathies [122]. Although the number of clinical studies evaluating topical capsaicin in the treatment of neuropathic pain caused by spine diseases is limited, their results are promising. A prospective open-label clinical trial reported a significant alleviation of neuropathic pain and improvement in the quality of life of patients with lumbosacral neuropathic pain after the administration of an 8% capsaicin patch, compared with placebo [171]. Moreover, a significant decrease in neuropathic pain after the use of an 8% capsaicin patch has also been observed in patients with low back radiculopathies [172]. However, further randomized studies are necessary to verify the effectiveness of topical capsaicin for spinal neuropathic pain and to compare it with other treatment modalities.

Botulinum toxin A (BTX-A), a potent neurotoxin, is currently recommended as a second-line treatment for neuropathic pain [104]. Changes provoked by subcutaneously injected BTX-A include the modulation of both peripheral and central sensitizations responsible for the neuropathic pain pathomechanism [127]. The clinical effectiveness of BTX-A was demonstrated in several randomized controlled trials in the treatment of neuropathic pain forms, such as trigeminal neuralgia, postherpetic neuralgia, diabetic peripheral neuropathy and pain after spinal cord injury [123,124,125,126]. Moreover, BTX-A injections demonstrated better effects in the treatment of chronic low back pain compared with saline injections, a combination of lidocaine and corticosteroids, and traditional acupuncture [173]. In a recent prospective open-label study, BTX-A injection significantly improved pain scores measured in patients with resistant chronic low back pain with mild transient complications in a minority of patients [128]. The above findings should be confirmed by well-designed studies with a larger sample size.

#### 5.1.4. Opioids

Opioids such as tramadol, tapentadol and oxycodone have also demonstrated potential effectiveness in relieving neuropathic pain with better results in peripheral than central neuropathic pain [112,129]. The high risk of misuse, addiction and morbidity comprise the main concerns of this class of drugs [131]. Tramadol, a µ-opioid agonist and 5-HT reuptake inhibitor, has been recommended as second-line treatment [108,112,141]. Somnolence and confusion may occur during treatment with tramadol, however, the risk of abuse is lower than other opioids [130]. Tapentadol, another µ-opioid agonist, has shown beneficial outcomes in the management of diabetic neuropathy and low back pain with a neuropathic component [174,175,176,177]. Other strong opioids such as oxycodone or morphine are recommended in the third-line treatment of neuropathic pain, but these agents are rarely utilized due to their high addiction potential [112]. A recent Bayesian network meta-analysis based on 2933 patients from published randomized controlled trials demonstrated that tapentadol, oxymorphone and fentanyl are the most effective opioids for pain symptoms in patients with chronic low back pain [178]. However, tramadol was not considered in this meta-analysis.

### 5.2. Neurostimulation Techniques

Long-term pharmacologic treatment often leads to developing tolerance and resistance to used medications. In the case of patients with neuropathic pain refractory to currently recommended pharmacologic treatment, CNS stimulation techniques may be a valuable therapeutic approach. These methods include spinal cord stimulation (SCS), dorsal root ganglion stimulation (DRGS), transcranial direct current stimulation (tDCS) and repetitive transcranial magnetic stimulation (rTMS). According to current French guidelines, SCS and rTMS are considered as a third-line treatment, whereas other neuromodulatory methods are not included [104] (Table 3).

#### 5.2.1. Spinal Cord Stimulation

Among the investigated neuromodulatory techniques, spinal cord stimulation (SCS) constitutes a well-established method, which has demonstrated potential effectiveness in the treatment of numerous diseases such as peripheral diabetic neuropathy, failed back surgery syndrome, irritable bowel syndrome, painful radiculopathy, complex regional pain syndrome and postherpetic neuralgia [179,182,183,184,185,186]. Moreover, the cost-effectiveness of this method compared with conventional treatment modalities has been demonstrated [201].

In the SCS technique, multiple electrodes (usually between 4 and 16) are implanted percutaneously or through the laminotomy into the epidural space nearby the posterior columns of the spinal cord [202]. Thereafter, a low-voltage electrical current is administered through inserted electrodes at a frequency of about 50–60 Hz [108]. The electrical stimulation alleviates the symptoms of neuropathic pain due to the interruption of the nociceptive transmission to the brain through the inhibition of the C and Aδ-fibers [187]. Apart from neurophysiological mechanism, SCS also acts through an increase in GABA and serotonin levels and a subsequent decrease in the release of glutamate and aspartate, which also contributes to the analgesic effect exerted by SCS [188].

However, stimulation of the afferent sensory fibers with a large diameter such as Aδ-fibers may induce paresthesia. Therefore, numerous modifications of the SCS technique have been investigated with different stimulation parameters, which may prevent SCS-induced paresthesia [141,203]. One of them, a burst SCS, which relies on the administration of five intermittent electrical pulses at a frequency of around 500 Hz, effectively alleviated the pain caused by failed back surgery syndrome and painful diabetic neuropathy, and significantly decreased paresthesia compared with tonic SCS [204]. Based on the results of a systematic review, a I/II level of evidence was determined for the efficacy of SCS, and a II/III level of evidence for high-frequency stimulation in the treatment of lumbar FBSS [205]. Moreover, SCS significantly reduces chronic low back pain regardless of a history of previous spine surgery [202,206].

Due to the invasiveness of the epidural implantation of electrodes, numerous complications are associated with SCS, including pain at the generator site (12% of cases), infection (4.5% of cases), nerve injury and epidural hematoma [112,180,189]. Moreover, migration, breakage, disconnection or failure of the electrode may complicate the treatment by SCS [181].

Nevertheless, SCS represents a relatively well-tolerated, effective, cost-efficient and reversible therapeutic modality for chronic neuropathic pain resistant to pharmacological treatment. In further studies, the optimal frequency of administered current should be established to maximize the effectiveness of the SCS technique.

#### 5.2.2. Dorsal Root Ganglion Stimulation

Dorsal root ganglion stimulation (DRGS) is a novel, more focused neuromodulatory technique for the treatment of neuropathic pain syndromes compared with standard SRS, enabling the inhibition of pain with even sub-dermatomal accuracy [207]. Moreover, DRGS may require less energy for stimulation and induce postural-independent paresthesia due to the lack of a CSF layer between electrodes and the stimulated dorsal root ganglion. On the other hand, the CSF layer present between SCS electrodes and nerve fibers results in an urgent need for higher current intensity and variable intensity of paresthesia due to the position-dependent thickness of the CSF layer [207]. Contrastingly, the implantation of a DRGS device is more complicated than SCS implantation. Regarding the site of DRGS lead implantation, the T12 nerve root seems to be an optimal location to treat low back pain [208]. DRGS has demonstrated beneficial outcomes in the treatment of discogenic low back pain, failed back surgery syndrome, phantom limb pain, complex regional pain syndrome and postherniorrhaphy groin pain [190,191,192,193,194].

In a multicenter randomized comparative trial, ACCURATE, the use of DRGS showed better outcomes in pain relief and resulted in less postural-dependent paresthesia compared with SCS in 152 patients diagnosed with complex regional pain syndrome and causalgia [209]. A currently recruiting BOOST-DRG study will compare the effectiveness of SCS, DRGS and a combined approach in the treatment of refractory chronic lower limb neuropathic pain and low back neuropathic pain (NCT04852107).

However, the long-term results of DRGS application for neuropathic pain and its cost-effectiveness remain unknown. Nevertheless, DRGS represents another promising method for the alleviation of drug-resistant neuropathic pain.

#### 5.2.3. Non-Invasive Brain Stimulation

Non-invasive brain stimulation (NIBS) techniques, such as transcranial direct current stimulation (tDCS) and repetitive transcranial magnetic stimulation (rTMS), commonly used in the treatment of psychiatric disorders, have recently been proposed for the management of neuropathic pain. In the vast majority of studies on NIBS, the primary motor cortex area (M1) constitutes the anatomical target for attenuating neuropathic pain. However, non-motor brain areas, such as the dorsolateral prefrontal cortex (DLPFC), premotor cortex (PMC), secondary somatosensory cortex (S2) and frontal cortex, have also been demonstrated as promising neurostimulation targets [210]. In both methods, the motor cortex is transcranially stimulated by an electric current, which may be induced by magnetic stimulation in the case of rTMS or by electric stimulation (1–2 mA) in the case of tDCS [211]. The procedure induces the release of striatal dopamine in the brain cortex, which may contribute to the analgesic effect of NIBS [196]. NIBS is generally considered a well-tolerated method, although some mild complications, such as local headache, tinnitus, burning, and in some cases, seizures, have been observed [197]. Moreover, in patients with aneurysm clips, cochlear implants, deep brain electrodes, epilepsy history and cardiac stimulators, rTMS should not be used [1].

rTMS represents the only NIBS technique approved for clinical use by the Federal Drug Administration with indications for drug-resistant major depression [212,213]. Numerous studies have proven effectiveness of rTMS targeting the M1 area of the motor cortex, also in the treatment of neuropathic pain with a longstanding analgesic effect [214,215,216,217]. A significant improvement in pain intensity has been observed in patients with chronic low back pain treated with rTMS without severe complications, compared with patients treated with physiotherapy and a sham group [218]. A currently recruiting randomized controlled trial will evaluate the effectiveness of rTMS alone and in combination with motor control exercises in relieving chronic low back pain (NCT04555278).

One tDCS technique has shown beneficial outcomes in alleviating neuropathic pain associated with traumatic spinal cord injury, stroke, multiple sclerosis, fibromyalgia, trigeminal neuralgia and diabetes [198]. A randomized controlled trial conducted by Straudi et al. showed significantly reduced pain and increased psychological well-being after the use of tDCS in patients with chronic low back pain compared with a sham-tDCS group [219]. However, a recent meta-analysis did not support these findings and demonstrated non-significant pain reduction after tDCS in patients with non-specific chronic low back pain [220]. A recently completed randomized controlled trial, conducted on 60 patients with chronic non-specific low back pain, evaluated the combined use of tDCS and therapeutic exercise (NCT03503422). However, its results have not been posted.

Nevertheless, NIBS techniques represent a non-invasive and potentially effective therapeutic option for neuropathic pain. However, the exact analgesic mechanisms of NIBS should be investigated in further studies, and multicenter randomized studies should be conducted to enhance clinical evidence for the effectiveness of NIBS [197].

#### 5.2.4. Optogenetic Stimulation

Optogenetic stimulation is a relatively new yet promising therapeutic method that involves affecting (both inhibiting and activating) proteins with light. Specific wavelengths affect light-sensitive transmembrane channels, changing their functioning. One of the regions involved in neuropathic pain is the anterior cingulate cortex (ACC), which is located in the limbic system; however, its detailed role remains unclear [216]. The optogenetic activation of dopamine receptors D1 and D2 in ACC may alleviate pain sensations [217].

### 5.3. Emerging Therapeutic Approaches

#### 5.3.1. Cannabis-Based Medicines

Recently, cannabis-based medicines have been proposed as a therapeutic option for neuropathic pain due to their potential analgesic properties. However, the evidence for the effectiveness of cannabis-based medicines in reducing the chronic neuropathic pain presented by a Cochrane systematic review was of a very low quality [133]. Moreover, most studies investigating cannabinoids in the management of neuropathic pain are characterized by a short follow-up [132]. Thus, long-term studies are indicated to further evaluate the effects of medical cannabis.

The most common adverse effects are transient and mild, and they include somnolence, confusion, dizziness, headaches, dry mouth, diarrhea, constipation and impaired neurocognitive performance [132]. In rare cases, acute psychosis has been observed [134]. Numerous studies have demonstrated that higher THC concentrations more often induce adverse effects than lower concentrations [221,222], whereas formulations with lower THC doses appear to be non-inferior compared with formulations with higher THC concentrations. Therefore, the use of lower THC concentrations (about 1.29%) is sufficient to obtain the analgesic properties in the treatment of neuropathic pain, and suggested to minimize adverse effects.

Nevertheless, further studies are necessary to precisely evaluate the potential advantages and disadvantages of medical cannabis in the treatment of neuropathic pain.

#### 5.3.2. Stem Cell Therapy

Stem cell transplantation (SCT), as a neuroregenerative therapy, has demonstrated the potential capability to regenerate nervous system injuries, such as spinal cord injury, multiple sclerosis and neurodegenerative disorders [223,224,225,226]. Recent studies indicated that SCT may also be an alternative therapeutic modality for neuropathic pain syndromes [200].

SCT can potentially alleviate neuropathic pain through anti-inflammatory action, the suppression of central sensitization and the inhibition of glial cell activation [200]. In numerous studies on animal models, the potential effectiveness of SCT for neuropathic pain has been proven [227,228,229]. A clinical trial by Vaquero et al. demonstrated that therapy with intrathecally administered mesenchymal stromal cells (MSCs) significantly and progressively decreased neuropathic pain in patients after spinal cord injury (*p* < 0.003) [199]. Moreover, intradiscally injected stem cells have demonstrated beneficial outcomes in the treatment of discogenic low back pain in several clinical studies [230,231,232]. However, the exact SCT mechanism of action remains unclear and should be explored in further preclinical studies. More research is needed to verify the effectiveness of SCT in relieving neuropathic pain and discogenic low back pain.

#### 5.3.3. Targeted Anti-Inflammatory Agents

Recent evidence indicates that inflammation significantly contributes to the pathomechanism of disorders causing neuropathic pain [233] In this phenomenon, numerous inflammatory cells and regulatory molecules are involved, including cytokines, non-coding RNAs, macrophages, T cells, cytokines and chemokines [233]. Therefore, searching for novel therapeutic agents targeting the inflammatory molecules contributing to the neuropathic pain mechanism is relevant.

Numerous anti-inflammatory agents, such as TNF-α, IL-1β, IL-6 and IL-17 inhibitors, have been tested for this purpose with variable outcomes [234] The increasing number of studies suggests an important regulatory role of non-coding RNAs, such as microRNAs or circRNAs, in the neuroinflammatory processes. This finding creates opportunity for their utilization as therapeutic targets or novel biomarkers for monitoring the effectiveness of neuropathic pain treatment [235]. However, the complexity of the inflammatory microenvironment may hamper the effectiveness of immune-targeted therapeutic strategies. Thus, a deep understanding of molecular patterns in disorders associated with neuropathic pain is necessary to obtain successful outcomes.

Nevertheless, targeted anti-inflammatory agents represent a promising therapeutic option for neuropathic pain syndromes.

#### 5.3.4. Other Emerging Pharmacological Agents

While non-pharmacological methods of neuropathic pain treatment have been increasingly investigated in recent years, novel medicaments have also been extensively studied in preclinical or early clinical studies.

Angiotensin II type 2 receptors (ATR2) are expressed in human nociceptive sensory neurons and may contribute in neuropathic pain modulation [236]. The inhibition of ATR2 may result in analgesic effects, which has been confirmed by increasing clinical evidence regarding the effectiveness of ATR2 inhibitors in the management of neuropathic pain [237,238]. However, recent randomized controlled trials evaluating selective ATR2 inhibitor EMA401 in postherpetic neuralgia and painful diabetic neuropathy (NCT03094195 and NCT03297294) were prematurely terminated due to preclinical evidence of long-term hepatotoxicity [136].

Na_V_1.7 channels, a subtype of selective sodium channels expressed within the nociceptive neurons, may also contribute to neuropathic pain mechanisms [1]. The application of Na_V_1.7 antagonists such as TV-45070 and BIIB074 did not significantly decrease pain intensity in randomized controlled trials regarding their effectiveness in postherpetic neuralgia [137,138]. However, Na_V_1.7 needs further investigation for more clear evidence.

Other potentially effective pharmacological agents for the treatment of neuropathic pain include nerve growth antagonists, oxcarbazepine, flavonoids, selective serotonin reuptake inhibitors (SSRIs) and NMDA antagonists [239,240,241,242,243,244] Despite unclear or poor results of initial research concerning these agents, their therapeutic potential for neuropathic pain should be deeply evaluated in future studies.

## 6. Conclusions

Available epidemiological studies have demonstrated that a neuropathic component of pain in spine diseases constitutes a common phenomenon. The response of neuropathic pain to conventionally used analgesic drugs such as NSAIDs and opioids is limited. Therefore, the diagnosis of neuropathic pain in diseases of the spine plays a crucial role in the selection of effective therapy and contributes to better clinical outcomes. However, in the diagnosis of patients with spinal disorders, distinguishing between chronic nociceptive pain and neuropathic pain very often remains a challenge. Detailed anamnesis and medical examination constitute crucial factors in increasing the chance of a correct diagnosis. Moreover, various scales and questionnaires, including LANSS, NPQ, DN4 and painDETECT, have been developed to help assess pain in patients and diagnose neuropathic pain correctly.

A difficult diagnostic procedure, methodological variations in existing studies, and as yet not fully understood pathomechanisms of neuropathic pain mean that an effective therapeutic approach or scheme has not been established to date. French guidelines and recommendations of The Special Interest Group on Neuropathic Pain (NeuPSIG) may be useful tools for the management of neuropathic pain caused by spine diseases in clinical practice. According to current guidelines, pharmacotherapy remains a mainstay in first-line treatment for neuropathic pain. However, the mediocre long-term efficacy of pharmacological agents, such as SNRIs, gabapentinoids or TCAs, has led to the development of numerous novel non-pharmacologic approaches. These interventions include SCS, DRGS, NIBS and stem cell therapy. Moreover, novel pharmacologic agents have been extensively studied in recent years, including cannabinoids, ATR2 inhibitors and Na_V_1.7 inhibitors. However, most of them require further studies before being introduced into clinical practice. Therefore, to date, pharmacotherapy and spinal cord stimulation recommended by recent guidelines remain the most applicable methods of treatment. In the future, to facilitate the diagnosis and management of neuropathic pain, it is essential to identify precise diagnostic criteria and find methods that are less invasive and equally effective as a current treatment, as well as to avoid long-term complications.

## Figures and Tables

**Table 1:** Frequency of neuropathic pain in patients with spine disorders investigated by epidemiological studies.

Study	Spine Disease	Frequency of Neuropathic Pain (Number of Patients)
Kim et al. [94]	**Overall**	**36.4% (404)**
	Stenosis with instability	31.7% (64)
	Stenosis without instability	39.5% (219)
	Herniated lumbar disc	34.7% (119)
	Degenerative lumbar scoliosis	22.2% (2)
Park et al. [95]	**Overall**	**36.0% (31)**
	Radicular pain	63.4% (24)
	Neurogenic claudication	15.6% (7)
Yamashita et al. [13]	**Overall**	**53.5% (993)**
	Spondylotic myelopathy	77.3%
	Ligament ossification	75.7%
	Low back pain	29.4%
	Spondylolysis	40.4%
	Radiculopathy	56.9%
El Sissi et al. [14]	**Overall**	**55% (628)**

**Table 2:** A summary of currently available pharmacological agents for neuropathic pain.

Pharmacological Agent	Mechanism of Action	Clinical Effectiveness	Adverse Effects	Recommendation by FG [104]	References
Pregabalin	binding to the α2-δ subunit of presynaptic voltage-gated Ca^2+^ channels in the dorsal horn of the spinal cord	postherpetic neuralgia, spinal cord injury, phantom limb syndrome and diabetes-induced neuropathy	peripheral edema, somnolence, dizziness, xerostomia and obesity	Second-line treatment	[106,107,108]
Gabapentin				First-line treatment	
Mirogabalin		postherpetic neuralgia and diabetic neuropathic pain	somnolence, dizziness	Not recommended	[109,110]
Tricyclic Antidepressants	increasing the level of noradrenaline in the dorsal horn of the spinal cord and locus coeruleus	postherpetic neuralgia, central postpartum pain, pain after spinal cord injury, and nerve injury	cognitive impairment, walking disturbances, urinary retention, constipation, dry mouth, and orthostatic hypotension	First-line treatment	[105,108,111,112,113]
serotonin and norepinephrine reuptake inhibitors	increasing the level of noradrenaline in the dorsal horn of the spinal cord and locus coeruelus	fibromyalgia, diabetic neuropathy, and low back pain	lack of appetite, constipation, dry mouth, anxiety, hyperhidrosis and nausea	First-line treatment	[111,114,115,116,117]
Topical lidocaine	blocking voltage-gated Na+ channels	postherpetic neuralgia, postsurgical chronic neuropathic pain and diabetic peripheral neuropathy	skin irritation	First-line treatment	[118,119,120]
Capsaicin patches	modulation of transient receptor potential cation channel vanilloid subfamily member 1 (TRPV1)	diabetic peripheral neuropathy, postherpetic neuralgia and HIV neuropathies	skin irritation	Second-line treatment	[105,121,122]
Botulinum toxin A	modulation of both peripheral and central sensitizations	trigeminal neuralgia, postherpetic neuralgia, diabetic peripheral neuropathy and pain after spinal cord injury	transient injection site pain	Second-line treatment	[123,124,125,126,127,128]
Tramadol	µ-opioid agonist and 5-HT reuptake inhibitor	peripheral neuropathic pain	Somnolence, confusion, and the risk of misuse and addiction	Second-line treatment	[112,129,130,131]
Cannabis-based medicines	binding to the CB1 and CB2 receptors, COX-2 inhibition	low-quality evidence for chronic neuropathic pain	somnolence, confusion, dizziness, headaches, dry mouth, diarrhea, constipation, impaired neurocognitive performance and psychosis	Not recommended	[132,133,134,135]
EMA401	Angiotensin II type 2 receptor (ATR2) inhibition	postherpetic neuralgia and painful diabetic neuropathy	risk of long-term hepatotoxicity	Not recommended	[136]
TV-45070 and BIIB074	NaV1.7 inhibition	postherpetic neuralgia	local skin reactions where application site pain and pruritus (TV-45070); headache, pyrexia, nasopharyngitis, sleep disorder and tremor (BIIB074)	Not recommended	[137,138]

**Table 3:** A summary of currently available non-pharmacological therapeutic methods for neuropathic pain.

Therapeutic Approach	Mechanism of Action	Clinical Effectiveness	Adverse Effects	Recommendation by French Guidelines [104]	References
Spinal Cord Stimulation	inhibition of the C and Aδ-fibers, and an increase in GABA and serotonin levels in the spinal cord	peripheral diabetic neuropathy, failed back surgery syndrome, irritable bowel syndrome, painful radiculopathy, complex regional pain syndrome, postherpetic neuralgia and chronic low back pain	pain at the generator site, infection, nerve injury, epidural hematoma, hardware failures	Third-line treatment	[112,179,180,181,182,183,184,185,186,187,188,189]
Dorsal Root Ganglion Stimulation	inhibition of the C and Aδ-fibers, and an increase in GABA and serotonin levels in the dorsal horn of the spinal cord	discogenic low back pain, failed back surgery syndrome, phantom limb pain, complex regional pain syndrome and postherniorrhaphy groin pain	lead fractures, nerve injury	Not recommended	[190,191,192,193,194,195]
Repetitive Transcranial Magnetic Stimulation	inducing the release of striatal dopamine in the brain cortex through magnetic stimulus	pain after spinal cord injury, phantom limb pain, pain secondary to malignancy, postherpetic neuralgia, diabetic neuropathy, chronic low back pain	headache, tinnitus, burning and seizures	Third-line treatment	[141,196,197]
Transcranial Direct Current Stimulation	inducing the release of striatal dopamine in the brain cortex through electric stimulus	traumatic spinal cord injury, stroke, multiple sclerosis, fibromyalgia, trigeminal neuralgia and diabetes	headache, tinnitus, burning and seizures	Not recommended	[196,197,198]
Stem Cell Therapy	anti-inflammatory effect, suppression of central sensitization and inhibition of glial cell activation	uncertain evidence for neuropathic pain after spinal cord injury	fever, headache, infections, potential cancerogenicity	Not recommended	[199,200]

## Data Availability

Not applicable.
